# A sugarcane R2R3-MYB transcription factor gene is alternatively spliced during drought stress

**DOI:** 10.1038/srep41922

**Published:** 2017-02-07

**Authors:** Jinlong Guo, Hui Ling, Jingjing Ma, Yun Chen, Yachun Su, Qingliang Lin, Shiwu Gao, Hengbo Wang, Youxiong Que, Liping Xu

**Affiliations:** 1 Key Lab of Sugarcane Biology and Genetic Breeding, Ministry of Agriculture, Fujian Agriculture and Forestry University, Fuzhou 350002, Fujian, China

## Abstract

MYB transcription factors of the R2R3-MYB family have been shown to play important roles in many plant processes. A sugarcane R2R3-MYB gene (*ScMYB2*) and its two alternative forms of transcript (*ScMYB2S1* and *ScMYB2S2*) were identified in this study. The deduced protein of *ScMYB2S1* is a typical plant R2R3-MYB protein, while *ScMYB2S2* encodes a truncated protein. Real-time qPCR analysis revealed that *ScMYB2S1* is suppressed under PEG-simulated drought stress in sugarcane, while *ScMYB2S2* is induced at later treatment stage. A senescence symptom was observed when *ScMYB2S1* was injected into tobacco leaves mediated by *Agrobacterium*, but no symptom for *ScMYB2S2*. Further investigation showed that the expression levels of 4 senescence-associated genes, *NtPR-1a, NtNYC1, NtCAT3* and *NtABRE*, were markedly induced in tobacco leaves after *ScMYB2S1-*injection, while they were not sensitive to *ScMYB2S2-*injection. Moreover, MDA and proline were also investigated after injection. Similarly, MDA and proline levels were induced by ABA and *ScMYB2S1*, while inhibited by *ScMYB2S2.* We propose that *ScMYB2*, by alternatively splicing two transcripts (*ScMYB2S1* and *ScMYB2S2*), is involved in an ABA-mediated leaf senescence signaling pathway and play positive role in respond to drought-induced senescence in sugarcane. The results of this study provide information for further research in sugarcane stress processes.

Transcription factors are proteins that bind to specific DNA sequences^1^, thereby promoting or blocking the recruitment of RNA polymerase to specific genes^2^,^3^. A typical plant transcription factor contains a DNA-binding region, an oligomerization site, a transcription-regulation domain, and a nuclear localization signal^4^,^5^. According to the structural features of the DNA-binding domain, plant transcription factors can be divided into several families^6^, and many of them, including MYB/MYC, AP2/EREBP, bZIP, NAC and WRKY, have been implicated in abiotic stress tolerance^7^,^8^.

MYB transcription factors are defined by a highly conserved MYB DNA-binding domain (DBD) at the N-terminus, and have been found in a wide variety of eukaryotic organisms, including animals, plants, insects and fungi^9^,^10^. Animal MYB proteins are referred to as 3R-MYB for their DBDs, which generally consists of three tandem amino acid sequence repeats (motif, designated R1, R2, and R3) of about 50~53 amino acid residues in length, and forms a helix-turn-helix fold with three regularly spaced tryptophan residues^11^,^12^. Plant MYB transcription factors can be classified into four major groups based on the number and position of adjacent MYB motif repeats, namely 1R-MYB (R1-MYB), 2R-MYB (R2R3-MYB), 3R-MYB (R1R2R3-MYB) and 4R-MYB (R1R2R2R1/2)^13^,^14^, containing one, two, three and four MYB repeats, respectively^15^. In plants, R2R3-MYB genes are predominant^16^,^17^, and members of this family function in a variety of plant-specific processes^18^, as evidenced by their extensive functional characterization in *Arabidopsis*^14^.

As one of the largest plant transcription factor families, MYB proteins are known to play key roles in gene transcriptional regulatory networks that mediate a variety of developmental processes and defense responses, including cellular differentiation^19^,^20^,^21^, morphogenesis^22^, light-signaling pathways^23^, secondary metabolism^24^, hormone signal transduction^25^, disease resistance and abiotic stress tolerance^26^,^27^. The regulatory activities of plant MYB proteins are elaborately regulated at multiple steps^14^,^28^. In particular, accumulating evidence illustrates that post-transcriptional control of mRNA modulates the transcription factor activities during plant response to environmental stimuli^28^. Alternative splicing (AS), to regulate gene expression, is one subset of post-transcriptional processes^29^. Through effecting the production of mRNA isoforms with different exonic composition from a single gene, alternative splicing creates multiple mRNA transcripts^30^. Alternative splicing can affect protein function and influence protein diversity when AS events occur within the translated regions of mRNAs^30^. It is also common for AS to result in isoforms that contain a premature termination codon, which subsequently become targets for nonsense mediated decay^30^. By this way, instead of a truncated polypeptide, no protein is produced.

Plant leaf senescence is an age-dependent deterioration process and is also triggered by environmental stresses and phytohormones^31^,^32^. It has been recognized that senescence associated genes (SAGs) are induced by senescence^32^,^33^. Abscisic acid (ABA) is an important phytohormone and plays a critical role in regulating plant development and responses to various stress signals, such as drought^32^. It is also well-known that ABA promotes leaf senescence^32^.

In the present work, the function of a sugarcane (*Saccharum officinarum*) R2R3-MYB gene *ScMYB2* with its two alternative splicing transcripts, *ScMYB2S1* and *ScMYB2S2I*, were investigated. We utilized real-time qPCR to evaluate the response of the alternative splicing transcripts to drought stress induced by PEG. A visible symptom of leaf senescence, i.e. de-greening, was observed when one alternatively spliced transcript of *ScMYB2S1* was transiently over-expressed in tobacco (*Nicotiana tabacum*) leaves. To further investigate the role of *ScMYB2S1* and *ScMYB2S2* in senescence, the contents of malonaldehyde (MDA) and proline, and then the expression profiles of 4 previously reported senescence-associated genes (SAGs) are checked in tobacco leaves after *ScMYB2S1*- or/and *ScMYB2S2*-transiently-transformed. Results suggest that *ScMYB2* is involved in the response to drought. The differential expression of two alternatively spliced transcripts during PEG stress could be one kind of drought tolerance molecular mechanism in sugarcane.

## Results

### Cloning and sequence analysis of *ScMYB2*

**Figure 1:**
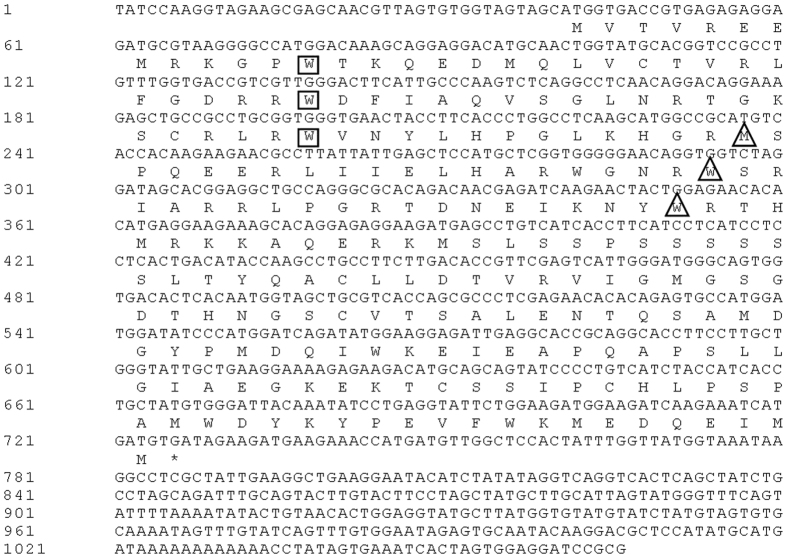
The nucleotide acid sequence and deduced amino acid sequence of *ScMYB2S1* gene. The “◻” shows the conservative tryptophan residual existed in R2 repeat of ScMYB2S1, and the “▵” shows the conservative tryptophan residual or other replaced amino acids existed in R3 repeat of ScMYB2S1.

Two rounds of PCR were performed, and gel electrophoresis of the inner PCR products showed one fragment (Supplementary Fig. S1). The amplicons of about 1,100 bp long were separated and recovered from the agarose gel, which were subsequently used for T-A cloning and transformed into *Escherichia coli*. Ten randomly positive clones were picked and sequenced, and two R2R3-MYB-like cDNA sequences were obtained, designated as *ScMYB2S1* (GenBank Accession Number KM387410) and *ScMYB2S2* (GenBank Accession Number KM387411), respectively. *ScMyB2S1* had a full length of 1, 066 bp, with an ORF of 687 bp, 5′ UTR (untranslated region) of 40 bp, and 3′UTR of 339 bp (Fig. 1). The deduced protein of *ScMYB2S1* was a typical plant R2R3-MYB protein, containing two MYB DNA-binding domains (R2 and R3 repeats) at the N-terminal. Within the R2 and R3 repeats, the highly conserved tryptophan (W) residues implicated in DNA-binding were spaced by the 19 or 18 amino acid residues, respectively. The first W of R3 repeat in ScMYB2S1 protein was replaced by a methionine (M) (Fig. 1).

**Figure 2:**
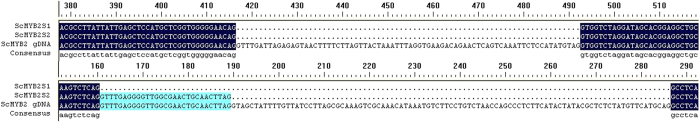
The analysis of intron loss in cDNA and gDNA of the *ScMYB2*.

Overlapping the full-length sequences of *ScMYB2S1*, 968-bp long *ScMYB2S2* transcript contained an additional 29-bp-sequence inserting into the corresponding location of the ORF, which interrupted the reading frame of a subsequent region behind the start codon and caused frameshift mutation of the sequence. Thus, compared with the amino acid sequence of ScMYB2S1, the first MYB DNA-binding domain (R2) in the amino acid sequence of ScMYB2S2 was missing, which resulted in the residue part starting with the first methionine (M) of R3 repeat, thereafter sharing 100% homology to the ScMYB2S1. Cloning a genomic sequence of the gene was also performed to identify whether the two transcripts, *ScMYB2S1* and *ScMYB2S2*, were produced by alternative splicing of the same gene. The genomic sequence of the *ScMYB2* gene (GenBank Accession Number KM387409) displayed at least two alternatively spliced isoforms (Fig. 2): a typical plant R2R3-MYB transcription factor gene *ScMYB2S1* and *ScMYB2S*2 encoding a truncated protein starting at a methionine in the R3 repeat.

The genomic sequence of the *ScMYB2* had a highly conserved splicing arrangement with three exons and two introns (126 bp and 76 bp). The 126 bp intron appeared to consist of two short tandem intron-like sequences, 29-bp sequence mentioned above at the 5′-terminal and the other 97 bp sequence at the 3′-terminal. All of them conformed to the GT-AG rule (Fig. 2).

**Figure 3:**
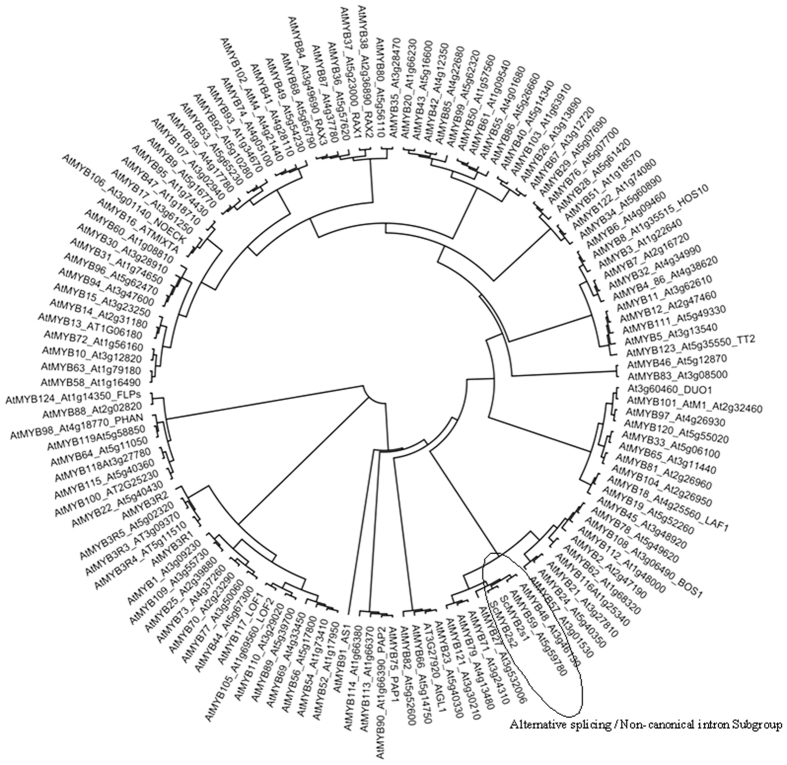
Phylogenetic relationships between *Arabidopsis* MYB transcription factors and ScMYB2S.

Following the methods described by Matus *et al*.^34^ and Lin-Wang *et al*.^24^, we constructed a phylogenetic tree with ScMYB2S1, ScMYB2S2 and the other 131 *Arabidopsis* MYB proteins using Mega5.05 software. Figure 3 indicated that ScMYB2S1 and ScMYB2S2 were close to AtMYB48 and AtMYB49, two members from *Arabidopsis* described as alternative splicing/non-canonical intron subgroup^34^.

### Expression profiles of *ScMYB2S1* and *ScMYBS2* under drought stress

**Figure 4:**
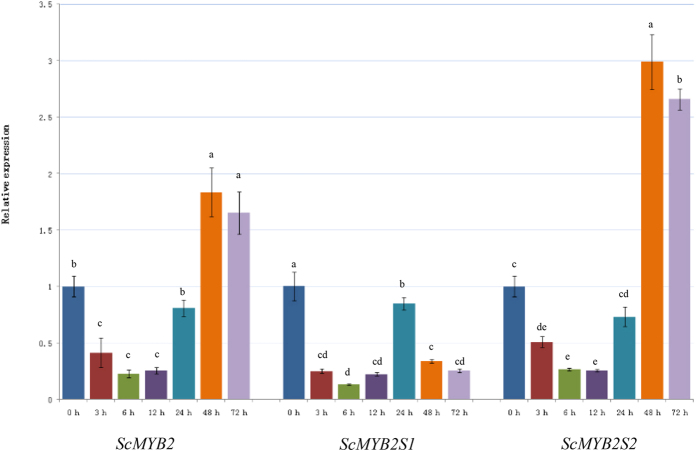
The expression profiles of *ScMYB2* and its two transcript versions under PEG -simulated drought stress. *ScMYB2* represents a pool of both variants. The other two are splice variant specific. Each value is the average of three replicate experiments ± standard error (n = 3). The different lowercase showed the significance difference of *ScMYB2, ScMYB2S1* and *ScMYB2S2* expression levels under p-value < 0.01 level.

To further examine the function of the alternatively spliced transcripts of *ScMYB2*, their responses to drought stress were performed. When treated with 25.0% PEG, the expression level of *ScMYB2* rapidly decreased at 3 h (Fig. 4) and stayed at the relatively low level during the periods from 3 h to 24 h, but increased in the later periods (48 h and 72 h). By using primers specific for each splice variant, the level of *ScMYB2S1* expression decreased steadily after 3 h following the treatment and maintained at a low expression level up to 72 h. In contrast, the expression of the *ScMYB2S2* increased dramatically at 48 and 72 h.

### *Agrobacterium*-mediated transient expression in tobacco leaves

**Figure 5:**
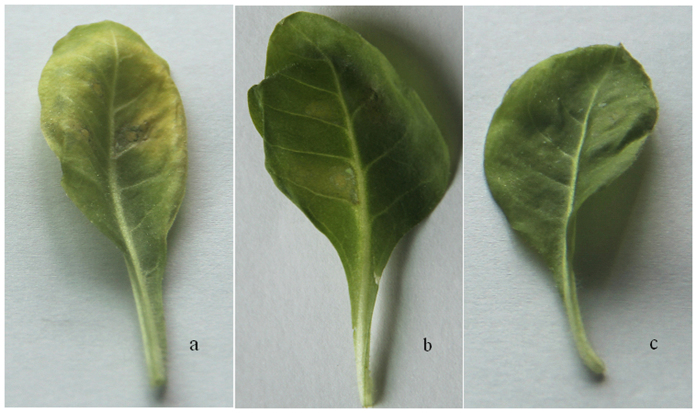
Phenotypic change of tobacco leaves after injection with different transcripts of *ScMYB2*. (**a**) pGreenII0229-ScMYB2S1; (**b**) pGreenII0229-ScMYB2S2; (**c**) Control: pGreenII0229.

To gain insights into the role of *ScMYB2* in sugarcane, transient expression of pGreenII0229-*ScMYB2S1*, pGreenII0229-*ScMYB2S2* and pGreenII0229 (control) were tested for their effect on tissue-cultured tobacco (*N. tabacum*) leaves via *A. tumefaciens* injection. The effect of over-expressing *ScMYB2S1, ScMYB2S2* or control was recorded at 24 h after injection. The whole leaf over-expressing *ScMYB2S1* changed color from green to yellow (Fig. 5a), when compared with control (Fig. 5c). Meanwhile, there was no obvious change in leaf color when the *ScMYB2S2* was over-expressed (Fig. 5b).

### Physiological measurement

**Figure 6:**
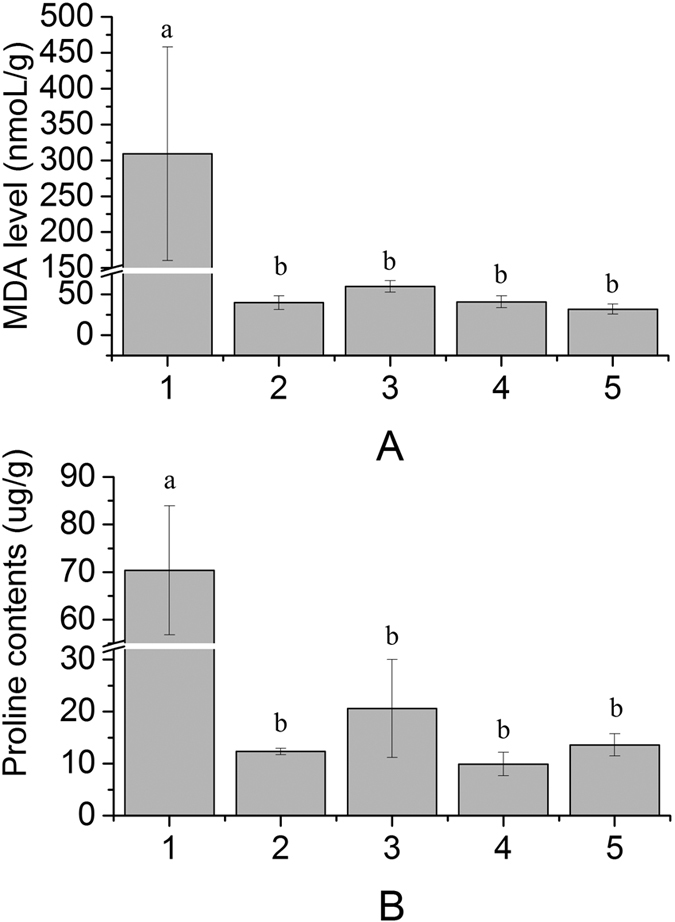
MDA and proline contents in tobacco leaves. 1: ABA-treatment; 2: Injected with recombinational *Agrobacterium* containing empty vector; 3. Injected with recombinational *Agrobacterium* containing the target gene of *ScMYB2S1*; 4. Injected with recombinational *Agrobacterium* containing the target gene of *ScMYB2S2*; 5: Injected with the mixture of the two recombinants containing *ScMYB2S1* and *ScMYB2S2.* (**A**) MDA level (nmoL/g); (**B**) Proline contents (μg/g). The different lowercase indicated the significance difference of MDA and proline contents under p-value < 0.01 level.

MDA and proline content in tobacco leaf samples were detected 48 h after ABA-treatment or injection. Figure 6 showed that *ScMYB2S*1-injection induced both MDA and proline levels obviously, while *ScMYB2S*2-injection and mixed-injection (with *ScMYB2S*1 and *ScMYB2S*2 volume ratio of 1:1) had limited effect on MDA or proline levels. Predictably, both MDA and proline contents were increased significantly after ABA-treatment (Fig. 6).

### Expression profiles of SAGs in tobacco leaves

**Figure 7:**
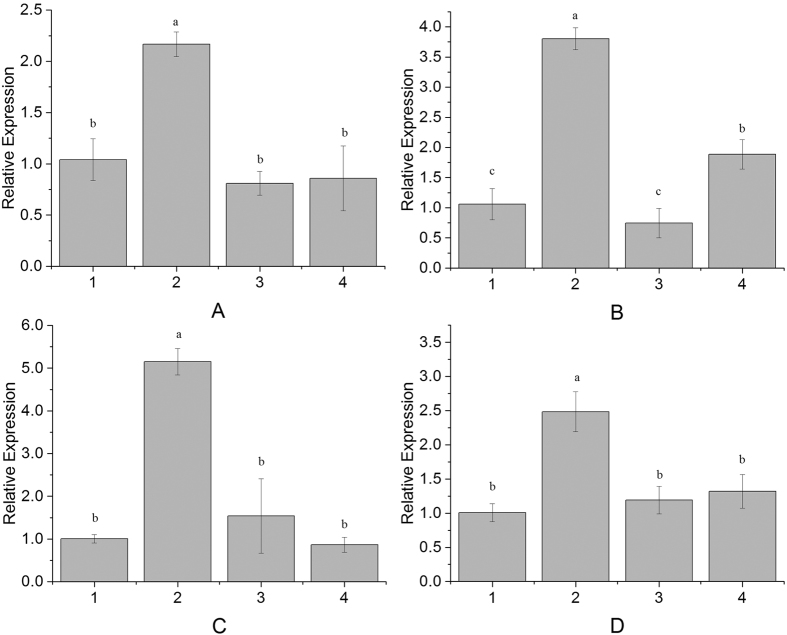
The expression profiles of 4 *SAGs* in tobacco leaves after injection. 1: Injected with recombinational *Agrobacterium* containing empty vector; 2. Injected with recombinational *Agrobacterium* containing the target gene of *ScMYB2S1*; 3. Injected with recombinational *Agrobacterium* containing the target gene of *ScMYB2S2*; 4: Injected with the mixture of the two recombinants containing *ScMYB2S1* and *ScMYB2S2*. (**A**) *NtNYC*; (**B**) *NtPR-1a*; (**C**) *NtCAT3*; (**D**) *NtABRE*. The different lowercase indicated the significance difference of 4 *SAGs* expression levels under p-value < 0.01 level.

Real-time qPCR was used to examine the expression profiles of four SAGs, *NtNYC1, NtPR-1a, NtCAT3* and *NtABRE*, in tobacco leaves under different treatments. All these genes were induced after *ScMYB2S1* injection, with an expression level about 2.17, 3.80, 5.14 and 2.48 times higher than that of the control, respectively (Fig. 7). Conversely, after injection of *ScMYB2S2*, the expression of *NtNYC1* and *NtPR-1a* were decreased with 0.81 and 0.75 times, respectively, lower than that of the control (Fig. 7). While at the same time, the expression level of *NtCAT3* and *NtABRE* were about 1.54 and 1.12 times higher than control (Fig. 6). Overall, however, the expression levels of these genes were between the two former situations after mix-injection of *ScMYB2S1* and *ScMYB2S2* (Fig. 7).

## Discussion

In the present study, a R2R3-MYB gene was isolated from sugarcane, designated as *ScMYB2*, showing to produce two alternatively spliced transcripts: *ScMYB2S1* and *ScMYB2S2*. Sequence analysis showed that the 126-bp-intron of the genomic sequence of the *ScMYB2* gene consisted of two short tandem “GT-AG” structures, which provided the structural basis for alternative splicing. Further sequence analysis revealed that ScMYB2S1 was a functional protein, containing two complete MYB DNA-binding domains (R2 and R3 repeats). ScMYB2S2, with the first MYB DNA-binding domain (R2 repeat) missing in the N-terminal amino acid sequence, might limited its function on directly involved in transcriptional regulation. Similarly, two *Arabidopsis* R2R3-type MYB genes, *AtMYB59* and *AtMYB48*, both were found to have four distinctively spliced transcripts that encoded either MYB-related proteins or R2R3-MYB proteins^35^. Interestingly, *ScMYB2, AtMYB59* and *AtMYB48* were found to be within the same phylogenetic subgroup in this study (Fig. 3).

Alternative splicing occurs widely in eukaryote and provides the main source of transcriptome and proteome diversity in an organism^36^. Recent studies suggested that the signal transduction related genes associated with various stress responses seemed to be particularly prone to alternative splicing in plants and animals^35^,^37^. The R2R3-MYB transcription factor superfamily has been showed to play an important role in many plant processes including abiotic stress responses^18^. It had been demonstrated that several MYB genes were alternatively spliced, and encoded proteins with regulatory functions^35^,^38^,^39^.

Previous studies have shown that alternative splicing also presents in sugarcane MYB transcription factor genes. Prabu *et al*.^40^,^41^ identified an inducible alternatively spliced R2R3-MYB transcription factor gene *ScMYBAS1* (EU670236) in *S. officinarum* based on a cDNA suppression subtractive hybridization library. Subsequently, semi-quantitative RT-PCR analysis revealed that the alternatively spliced transcripts, *ScMYBAS1-2* and *ScMYBAS1-3*, were constitutively expressed at high level when subjected to water deficit and salt stress treatments, however no sequence information was provided^40^,^41^. In GenBank the genomic DNA of *MYBAS1* (HM136779) from *Saccharum* hybrid cultivar Co740, and three alternatively spliced variants: *MYBAS1V1* (HM136780), *MYBAS1V*2 (HM136781) and *MYBAS1V*3 (HM136782), are noted as being induced by water deficit stress based on semi-quantitative RT-PCR analysis. Sequence analysis shows that *MYBAS1V1* is only 2 bases different from *ScMYBAS1* (Data not showed).

Many members in plant R2R3-MYB family have been reported as abiotic-stress-induced transcription factors^40^,^41^,^42^,^43^. Moreover, over-expressing the R2R3-MYB genes improved the resistance to freezing, drought, and salt stresses in transgenic plants^44^,^45^,^46^. In this study, *ScMYB2S2*, one alternatively spliced transcript of *ScMYB2*, was induced at the late periods of PEG-simulated drought stress. Contrary to expectation, the expression of *ScMYB2S1*, the other alternatively spliced transcript of *ScMYB2*, was suppressed constitutively by PEG-simulated drought stress. This shows that highly specific probes or primers are crucial to identify the expression pattern of a certain gene for alternatively spliced transcripts or allelic genes which are highly homologous to each other.

It was reported that leaf senescence was affected by drought stress^47^. In this study, the de-greening symptom displayed when one alternatively spliced transcript of *ScMYB2S1* was transiently over-expressed in tobacco leaves, suggesting the function of *ScMYB2S1* might relate to leaf senescence regulation. The stress hormone ABA promotes leaf senescence in an ethylene-independent pathway and induces compatible solutes (e.g. malondialdehyde and proline) accumulation in leaf^48^,^49^. Within those compatible solutes, MDA and proline levels are commonly known as markers of stress^50^. To further investigate the role of *ScMYB2S1* and *ScMYB2S2* in leaf senescence, the contents of MDA and proline in tobacco leaves were detected in this study. The results showed that both MDA and proline levels were significantly induced by ABA treatment (Fig. 6). Similarly, the two compatible solutes were also obviously increased after *ScMYB2S1*-injection (Fig. 6). On the contrary, *ScMYB2S2* -injection had less effect on them (Fig. 6). From those results, we can concluded that it is *ScMYB2S1* rather than *ScMYB2S2*, lead to the accumulation of MDA and proline in tobacco leaves.

Plant senescence is regulated by senescence associated genes (SAGs)^33^. Stress-induced senescence signals are perceived and then transferred via SAGs^51^. To date, a series of SAGs have been identified from various plant species^33^,^52^. In this study, 4 SAGs genes were selected randomly to investigate whether they were involved in the co-expression network regulating by *ScMYB2*. Among these four SAGs, pathogenesis-related 1a (PR1a) and catalase (CAT) have been identified as marker gene of biotic stress^51^. Non-yellow coloring (*NYC1*) gene, encoding the membrane-spanning isoform of Chl b reductase was expressed during leaf senescence in rice^53^, and its function on chlorophyll degradation also has been proved in *Arabidopsis*^33^. Recent studies have shown that abscisic acid responsive element (ABRE)-binding factor gene (ABRE) also involved in responding to ABA-induced leaf senescence^52^,^54^. An ABRE-binding factor has been identified as a positive regulator of abiotic stress and ABA signaling both in *Arabidopsis* and in rice^55^. Collectively, the function of all these four genes was identified to accelerate leaf senescence^33^,^56^. Previous studies have shown that both endogenous ABA and exogenously applied ABA can induce the expression of SAGs and promote leaf de-greening and senescence in general^52^. The present study showed that these 4 SAGs were significantly induced by *ScMYB2S1-*injection, while *ScMYB2S2*-injection didn’t have much effect (Fig. 7). We concluded that when transiently over-expressing in tobacco leaf, it is *ScMYB2S1* rather than *ScMYB2S2* playing an ABA-like function, induced the expression of the four SAGs.

*ScMYB2S1* was down-regulated in responding to drought stress during the whole processing period in sugarcane. Combined analysis of its performance in de-greening and promoting both compatible solute contents and the expression levels of SAGs in tobacco (Figs 5, 6 and 7) suggested that *ScMYB2S1* could act as a negative regulator and play a positive role in response to drought-induced senescence process. Meanwhile, the expression level of *ScMYB2S2* in sugarcane was up-regulated by drought stress at the later stage. It seems that *ScMYB2* tends to generate the isoform of *ScMYB2S2* by alternative splicing during the later stage. Different with *ScMYB2S1, ScMYB2S2* has limited effect on leaf de-greening (Fig. 5), MDA and proline contents (Fig. 6) and the expression of SAGs. It needs to further investigate whether this limited influence is due to its incomplete functional domain. And, recent studies have shown that alternative splicing of some transcription factor genes generates small interfering peptides (siPEPs), which negatively regulates the target transcription factors^28^. Further studies are also needed to substantiate whether the two transcripts are existed an interaction at nucleic acid level or protein level.

In conclusion, a hypothesis is proposed here that *ScMYB2*, by alternatively splicing two transcripts (*ScMYB2S1* and *ScMYB2S2*), might involve in an ABA-mediated leaf senescence signaling pathway and play positive role in respond to drought-induced senescence in sugarcane. The differential expression of two alternatively spliced transcripts during PEG stress could be one kind of drought tolerance molecular mechanism in sugarcane. The alternative splicing of the transcription factor gene *ScMYB2* may be pivotal to the molecular defense mechanism of sugarcane during drought-induced senescence processes.

## Methods

### Materials and treatments

3′-Full RACE Core Set Ver.2.0 Kit, TaKaRa LA PCR^TM^ *in vitro* Cloning Kit, PrimeScript RT-PCR Kit, DNA markers were purchased from TaKaRa (Dalian, China). RQ1 RNase-Free DNase was obtained from Promega Corporation (USA), SYBR^®^ Green PCR Master Mix Kit was purchased from Applied Biosystems TM (USA), and the instrument used in the real-time qPCR analysis was the ABI PRISM7500 real-time PCR system (USA). Plant Malondialdehyde (MDA) assay Kit and Proline assay Kit were purchased from Nanjing Jiancheng Bioengineering Institute (Nanjing, China).

Sugarcane cultivar Badila (*S. officinarum*), tobacco (*N. tabacum*) variety K326 seedlings and tissue culture plantlets used in this study was provided by the Key Laboratory of Sugarcane Biology and Genetic Breeding, Ministry of Agriculture, Fuzhou, China. According to Guo *et al*.^57^, uniform plantlets of an elite sugarcane cultivar Badila were grown in 1/4 Hongland nutrient solution for one week and then subjected to PEG8000 (25.0%) treatment. The sampling times were 0 h, 3 h, 6 h, 12 h, 24 h, 48 h and 72 h after the start of treatment.

*Agrobacterium*-infiltrated transient transformation of tobacco was carried out according to Lin-Wang described^24^. For phenotype observation assay, tobacco tissue culture plantlets were used for transiently transformed assay. Approximately 150 μL of each recombinant *Agrobacterium* culture was infiltrated at four points into a tender leaf. Tobacco seedlings grown in a greenhouse with environmental control systems were used for physiological measurement and gene expression assay. Two kind of stress treatments were applied to 8-week-old plants before flowering: ABA treatment (sprayed 100 μM ABA on leaf at 48 h) and *Agrobacterium* injection (using a suspension of recombinational *Agrobacterium* introduced into tobacco leaf by direct injection). As for the latter, approximately 300 μL of *Agrobacterium* containing the target gene or empty vector control were infiltrated at four points into the leaf. After 48 h treatment, all the leaf samples were collected and divided into three groups and assayed, respectively. One group was fixed in liquid nitrogen immediately and stored in a refrigerator at −85 °C until RNA extraction. According to the methods from Plant Malondialdehyde (MDA) assay Kit and Proline assay Kit, the other two groups were grinded in buffer solution immediately and then measured, respectively. All of the treatments were repeated independently three times. The data were analyzed with DPS v7.05 directly and the significance difference of the MDA and proline contents were marked using different lowercase in figure.

### Molecular cloning, sequencing and bioinformatics analysis

A sugarcane R2R3-MYB EST was obtained based on the bioinformatics analysis using the data from the previous RNA-Seq experiments (not yet published). Two nested gene-specific 3′ RACE primers was designed according to the EST sequence information, and one pair of gene-specific primer was designed to amplify the given target regions using genomic DNA as a template. Primer sequences are as follows:

3′ RACE GSP1: 5′-ACATAGTGGCTTCTTCTCCC-3′;

3′ RACE GSP2: 5′-TATCCAAGGTAGAAGCGAGCAA-3′;

ScMyb2 F: 5′-TATCCAAGGTAGAAGCGAGCAA-3′ (same to 3′ RACE GSP2);

ScMyb2 R: 5′-CCATAAGCATACCTCCAGTGTT-3′.

The method used in 3′ RACE was followed to the specifications of the 3′-Full RACE Core Set Ver.2.0 Kit (Takara). A full-length R2R3-MYB homolog gene of sugarcane (named *ScMYB2*) was identified by Blastx (http://blast.ncbi.nlm.nih.gov/Blast.cgi) with two Myb-like DNA-binding domains (pfam00249). The open reading frame (ORF) of the full-length cDNA sequence of *ScMYB2* was predicted using the ORF Finder online tool from NCBI (http://www.ncbi.nlm.nih.gov/gorf/gorf.html). Sequence alignment was performed using DNAMAN 5.2.2 software.

### Expression profiles of *ScMYB2* under PEG-simulated drought stress

Total RNA isolation was performed using the TRIzol^®^ Reagent (Invitrogen, USA). The removal of DNA from RNA samples was realized by RQ1 RNase-Free DNase (Promega, USA). The reverse transcription was realized by following the specifications of the PrimeScript^®^ RT reagent Kit (Takara, China). Finally, the SYBR^®^ Green PCR Master Mix (AB, USA) was employed in real-time qPCR reaction.

The *25 S* rRNA (BQ536525) gene was chosen as an internal control in real-time qPCR analysis^57^,^58^ and the forward and reverse primers for *25 S* rRNA were 5′-GCAGCCAAGCGTTCATAGC-3′and 5′-CCTATTG GTGGGTGAACAATCC-3′^58^. Based on the common sequences of *ScMYB2S1* and *ScMYB2S2*, a pair of real-time qPCR primers was designed using the Primer Express 3.0 software and the forward and reverse primers for *ScMYB2* were 5′-ACCGTCGTTGGGACTTCATT-3′ (MYB2qF) and 5′-CAGGCTTGGTATGTCAGTGAGGAG-3′ (MYB2qR), respectively (Fig. S2). Moreover, pairing with common reverse primer (MYB2qR), two sequence-specific forward primers were designed for *ScMYB2S1* and *ScMYB2S2* with the sequences 5′-CATTGCCCAAGTCTCAGGCC-3′ (MYB2S1qF) and 5′-CATTGCCCAAGTCTCAGGTT-3′ (MYB2S2qF), respectively (Supplementary Fig. S2).

The real-time qPCR reaction was realized with following conditions: 2 min at 50 °C, 10 min at 95 °C, and then 40 cycles of 94 °C for 15 s, and 60 °C for 60 s. Each assay was repeated by three times. When the reaction was completed, a melting curve was obtained. The 2^−ΔΔ^CT method was adopted to analyze the real-time qPCR results^59^. The data were analyzed with DPS v7.05 directly and the significance difference of the gene expression were marked using different lowercase in figure.

### Binary vectors construction

To study the function of *ScMYB2S1* and *ScMYB2S2* in tobacco, PCR was performed using the clone containing *ScMYB2S1* or *ScMYB2S2* as template to obtain the *ScMYB2S1* or *ScMYB2S2* ORF with matched sites. The primer sequences were MYB2S1F: 5-CCCAAGCTTATGGTGACCGTGAG-3, MYB2S2F: 5-CCCAAGCTTATGTCACCACAAGAA-3 and MYB2SR: 5-CGGAATTCTCACATCATGATTTCT-3′ (*Hin*d III and *Eco*R I sites are underlined). The 50 μL PCR reaction mix contained 5.0 μL 10 × PCR buffer; 4.0 μL deoxynucleotide triphosphates (dNTPs) (2.5 mM); 2.0 μL each of forward and reverse primers (10 μM); 2.0 μL plasmid DNA (100 ng); and 0.25 μL Ex-Taq enzyme (5 U/μL). The ddH_2_O was added as supplement. The PCR amplification program consisted of pre-denaturation for 5 min at 94 °C; denaturation for 30 s at 94 °C, annealing for 30 s at 55 °C, and extension for 45 s at 72 °C for 30 cycles; and final extension for 10 min at 72 °C. The *ScMYB2S1* or *ScMYB2S2* ORF with *Hin*d III and *Eco*R I sites was subcloned into intermediate vector pSIM35Scassett (*Hin*d III - *Eco*R I sites) in the *E. coli* strain DH5α to generate the putative recombinants. The intermediate vector containing the gene expression cassette of ‘35S promoter-ScMYB2S1 (or ScMYB2S2)-CaMV polyA’ was digested with *Eco*R V and these fragments were ligated into the equally digested *Eco*R V pGreen II0229 (Supplementary Fig. S3). A clone with a recombinant plasmid was validated by PCR, double digestion and sequencing, and was termed as pGII0229-ScMYB.

### *Agrobacterium*-mediated transient expression

A single colony of *A. tumefaciens* strain EHA105 was cultured overnight in 5 mL LB broth at 28 °C with 250 rpm shaking. Three mL of this culture was shifted to 1,000 mL LB medium and incubated at the same conditions until the OD_600_ was about 0.5. The culture was centrifuged at 3,500 × g for 15 min at 4 °C. The pellet was resuspended/washed with 15% glycerol in ultrapure water. After washing, the bacteria were pelleted again. All the steps were performed on ice. This process of washing was repeated three times. After final washing, the pellet was divided into 100 μL and stored at −80 °C.

Electroporation was carried out with an electric pulse of 2.0 kV/cm and 400Ω. After each transformation the bacterial cells were resuscitated by suspending in 1 mL of liquid LB media and subsequent incubation at 28 °C with 250 rpm shaking, before spreading on the LB plates. pGII0229, pGII0229-ScMYB2S1 and pGII0229-ScMYB2S2 were used for electroporation, respectively. Screening of positive clones was performed on LB medium containing 50 μg/mL kanamycin and 35 μg/mL rifampicin. The plasmid was isolated from the positive clones, and PCR was employed to confirm the transformation. *Agrobacterium*-infiltrated transient transformation of tobacco was carried out as previously described^24^.

### Expression profiles of SAGs in tobacco leaves

Four verified senescence-associated genes (SAGs), *NtPR-1a* (ACC. No. X12737.1)^51^, *NtCAT3* (Acc. No. Z36977.1)^51^, *NYC*1(XM016652882)^33^ and *NtABRE* (KF736850)^33^, were selected to verify their expression profiles in tobacco leaves after ABA treated or *ScMYB2s*-transiently-transformed, respectively. The forward and reverse primers for *NtPR-1a* were 5′-ATATCCCACTCTTGCCGTGCCCAA-3′ and 5′-GCTACCTGGTCGTCCCAGGTCAA-3′^51^. The forward and reverse primers for *NtCAT3* were forward 5′-CTGCAGCTCCCAGTTAAC-3′ and 5′-GAGCATCAACCCATCTGCA-3′^51^. Using the Primer Express 3.0 software, real-time qPCR primers were designed according to *NtNYC1* (ACC. No. XM016652882.1) and *NtABRE* (ACC. No. KF736850) sequences, respectively. The forward primers for *NtNYC1* and *NtABRE* were 5′-TGACATTTGGGTAAACAACGCT-3′ and 5′-CTAATAGGAACACGGGTGAAACTG-3′, respectively. The reverse primers for *NtNYC1* and *NtABRE* were 5′-ATCCATAGACAGCCGTTAGAGGG-3′ and 5′-ATCCATAGACAGCCGTTAGAGGG-3′, respectively. The elongation factor 1α (ELF-1α) (AF120093) gene was chosen as an internal reference gene for normalization of real-time RT-PCR in tobacco and the forward and reverse primers for EF-1a were 5′-TGAGATGCACCACGAAGCTC-3′and 5′-CCAACATTGTCACCAGGAAGTG-3′^60^. The real-time qPCR reaction was realized with following conditions: 2 min at 50 °C, 10 min at 95 °C, and then 40 cycles of 94 °C for 15 s, and 60 °C for 60 s. Each assay was repeated by three times. When the reaction was completed, a melting curve was obtained. The 2^−△△^CT method was adopted to analyze the RT-qPCR results^60^. The data were analyzed with DPS v7.05 directly and the significance difference of the gene expression were marked using different lowercase in figure.

## Additional Information

**How to cite this article**: Guo, J. *et al*. A sugarcane R2R3-MYB transcription factor gene is alternatively spliced during drought stress. *Sci. Rep.* **7**, 41922; doi: 10.1038/srep41922 (2017).

**Publisher's note:** Springer Nature remains neutral with regard to jurisdictional claims in published maps and institutional affiliations.

## Supplementary Material

Supplementary Figures
