# Supplementary material for: A sugarcane R2R3-MYB transcription factor gene is alternatively spliced during drought stress
Source: Sci Rep. 2017 Feb 7;7:41922. doi: 10.1038/srep41922 (PMC5294458; doi:10.1038/srep41922)
Supplement: Supplementary Figures [file srep41922-s1.pdf]

Supplementary Figures

A sugarcane R2R3-MYB transcription factor gene is alternatively spliced during drought stress

Jinlong Guo*, Hui Ling*, Jingjing Ma, Yun Chen, Yachun Su, Qingliang Lin, Shiwu Gao, Hengbo Wang, Youxiong Que & Liping Xu

Key Lab of Sugarcane Biology and Genetic Breeding, Ministry of Agriculture, Fujian Agriculture and Forestry University, Fuzhou 350002, Fujian, China

* These authors contributed equally to this work.

Correspondence and requests for materials should be addressed to L.X. (xlpmail@126.com)

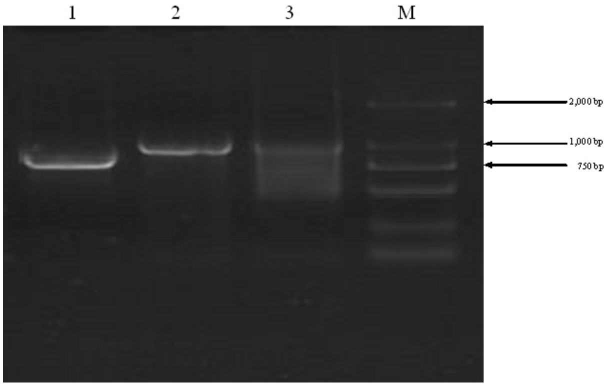

Supplementary Figure S1: 3'RACE inner PCR products of *ScMYB2*. 1: Control provided by the kit; 2: PCR products of *ScMYB2* (Using stem cDNA templates); 3: PCR products of *ScMYB2* (Using root cDNA templates); M: DL2,000 DNA Ladder.

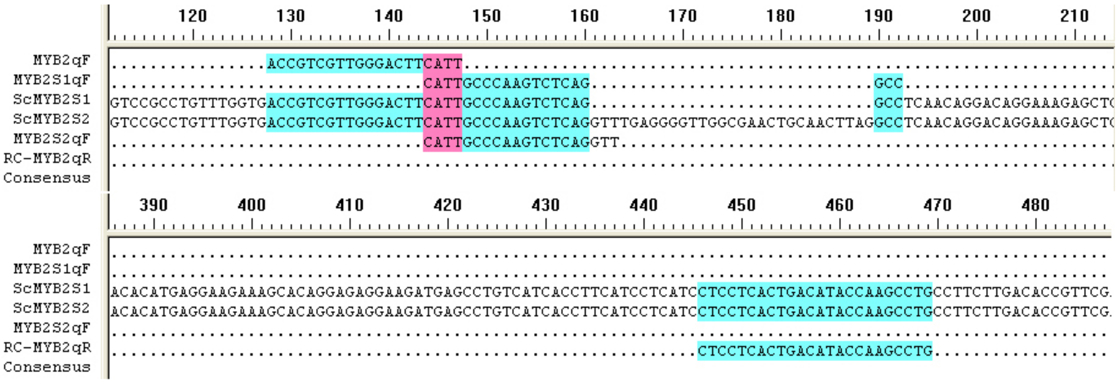

Supplementary Figure S2: Real-time qPCR primers for *ScMYB2*.

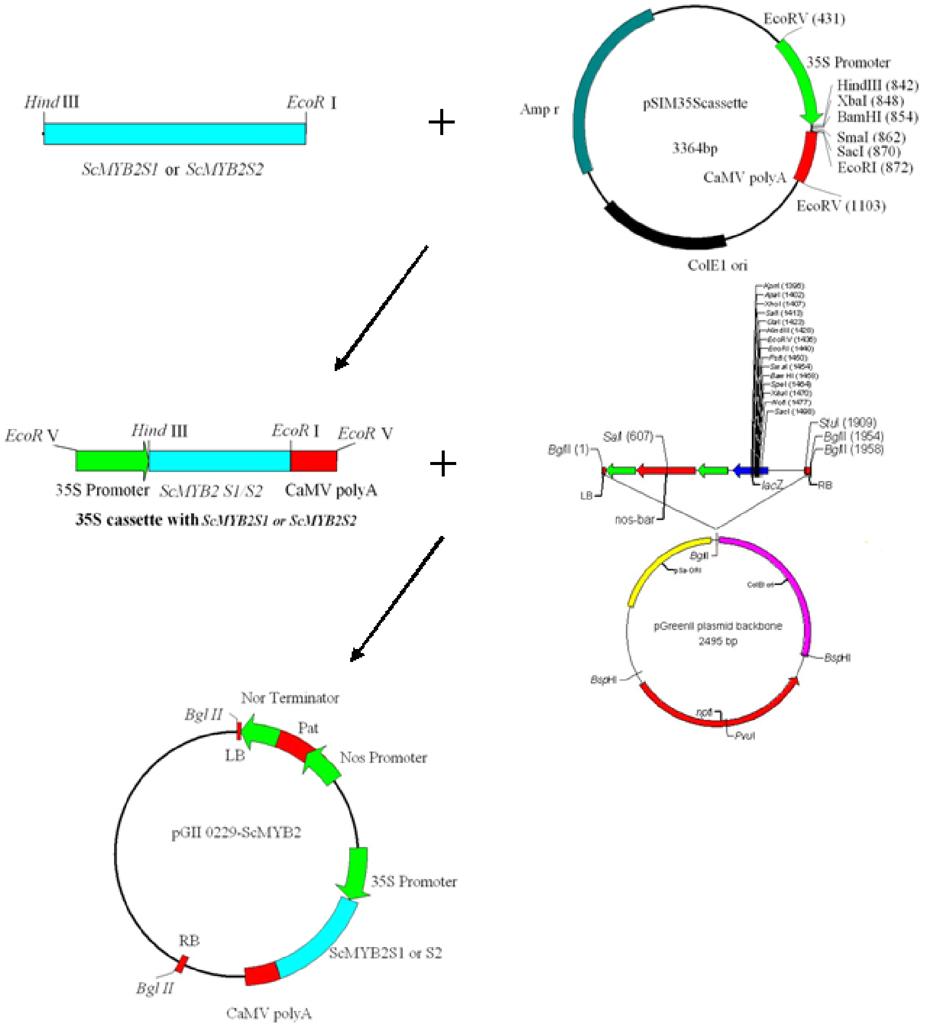

Supplementary Figure S3: A simplified flowchart showing the construction of binary vector.
